# The effects of long-term daily folic acid and vitamin B_12_ supplementation on genome-wide DNA methylation in elderly subjects

**DOI:** 10.1186/s13148-015-0154-5

**Published:** 2015-11-14

**Authors:** Dieuwertje E. G. Kok, Rosalie A. M. Dhonukshe-Rutten, Carolien Lute, Sandra G. Heil, André G. Uitterlinden, Nathalie van der Velde, Joyce B. J. van Meurs, Natasja M. van Schoor, Guido J. E. J. Hooiveld, Lisette C. P. G. M. de Groot, Ellen Kampman, Wilma T. Steegenga

**Affiliations:** Division of Human Nutrition, Wageningen University, PO Box 8129, 6700 EV Wageningen, The Netherlands; Department of Clinical Chemistry, Erasmus University Medical Center, Rotterdam, The Netherlands; Genetic Laboratory Internal Medicine, Erasmus University Medical Center, Rotterdam, The Netherlands; Department of Epidemiology, Erasmus University Medical Center, Rotterdam, The Netherlands; Department of Internal Medicine, Section of Geriatrics, Academic Medical Center, Amsterdam, The Netherlands; Department of Epidemiology and Biostatistics, EMGO Institute for Health and Care Research, VU University Medical Center, Amsterdam, The Netherlands

**Keywords:** DNA methylation, Folic acid, Vitamin B_12_, B-vitamins, One-carbon metabolism, Intervention trial, Infinium 450k BeadChip, Elderly, Cancer, Development, Epigenetics

## Abstract

**Background:**

Folate and its synthetic form folic acid function as donor of one-carbon units and have been, together with other B-vitamins, implicated in programming of epigenetic processes such as DNA methylation during early development. To what extent regulation of DNA methylation can be altered via B-vitamins later in life, and how this relates to health and disease, is not exactly known. The aim of this study was to identify effects of long-term supplementation with folic acid and vitamin B_12_ on genome-wide DNA methylation in elderly subjects.

This project was part of a randomized, placebo-controlled trial on effects of supplemental intake of folic acid and vitamin B_12_ on bone fracture incidence (B-vitamins for the PRevention Of Osteoporotic Fractures (B-PROOF) study). Participants with mildly elevated homocysteine levels, aged 65–75 years, were randomly assigned to take 400 μg folic acid and 500 μg vitamin B_12_ per day or a placebo during an intervention period of 2 years. DNA was isolated from buffy coats, collected before and after intervention, and genome-wide DNA methylation was determined in 87 participants (*n* = 44 folic acid/vitamin B_12_, *n* = 43 placebo) using the Infinium HumanMethylation450 BeadChip.

**Results:**

After intervention with folic acid and vitamin B_12_, 162 (versus 14 in the placebo group) of the 431,312 positions were differentially methylated as compared to baseline. Comparisons of the DNA methylation changes in the participants receiving folic acid and vitamin B_12_ versus placebo revealed one single differentially methylated position (cg19380919) with a borderline statistical significance. However, based on the analyses of differentially methylated regions (DMRs) consisting of multiple positions, we identified 6 regions that differed statistically significantly between the intervention and placebo group. Pronounced changes were found for regions in the *DIRAS3*, *ARMC8*, and *NODAL* genes, implicated in carcinogenesis and early embryonic development.

Furthermore, serum levels of folate and vitamin B_12_ or plasma homocysteine were related to DNA methylation of 173, 425, and 11 regions, respectively. Interestingly, for several members of the developmental *HOX* genes, DNA methylation was related to serum levels of folate.

**Conclusions:**

Long-term supplementation with folic acid and vitamin B_12_ in elderly subjects resulted in effects on DNA methylation of several genes, among which genes implicated in developmental processes.

**Electronic supplementary material:**

The online version of this article (doi:10.1186/s13148-015-0154-5) contains supplementary material, which is available to authorized users.

## Background

During early development, DNA methylation is one of the epigenetic phenomena responsible for programming of gene expression profiles [1]. It has been shown that the plasticity of epigenetic regulation can be determined by environmental factors, such as parental diet and lifestyle, during the critical windows in early development [1–3]. B-vitamins play an essential role in one-carbon metabolism and have, as such, also been implicated in the regulation of DNA methylation and DNA synthesis [1, 4–6].

Recently, neonatal folate-sensitive regions of differential methylation were identified based on the maternal folate status during the last trimester of pregnancy [7]. Maternal use of folic acid before and during pregnancy has been previously associated with specific DNA methylation patterns in the infants [8–10]. Also, maternal vitamin B_12_ levels have been shown to influence global DNA methylation in cord blood, whereas infants’ levels of vitamin B_12_ were associated with distinct gene-specific DNA methylation patterns [11]. These findings illustrate and confirm the potential role of B-vitamins on DNA methylation during early development [12, 13].

The “developmental origins of health and disease” hypothesis states that adult diseases often originate from aberrant epigenetic programming during early development [14, 15]. To what extent environmental exposures later in life can induce changes in DNA methylation and how this relates to development of adult diseases is an emerging field of research. Although some studies demonstrate plasticity of DNA methylation in adults [16, 17], it is still largely unknown to what extent B-vitamins involved in one-carbon metabolism can affect DNA methylation throughout the life cycle. Although not consistently, suboptimal levels of B-vitamins and homocysteine, a major key player in one-carbon metabolism, in adult life have been implicated in several clinical phenotypes [18], such as an increased risk of osteoporosis [19, 20], vascular diseases [21, 22], cognitive impairment [23], and cancer [24–26]. There is a growing body of evidence that the association between B-vitamins and disease risk implies a narrow range of an optimal B-vitamin status, and depends, in case of cancer, on the presence of preclinical neoplastic lesions [27–30]. Detailed insight into the effects of B-vitamins on DNA methylation is therefore urgently required in order to elucidate the mechanisms underlying the suggested effects of these B-vitamins on health and disease in adult life.

The aim of the current study was to determine the effects of a long-term daily supplementation with folic acid and vitamin B_12_ on genome-wide DNA methylation in leukocytes of elderly subjects.

## Results

### Biochemical analyses

**Fig. 1:**
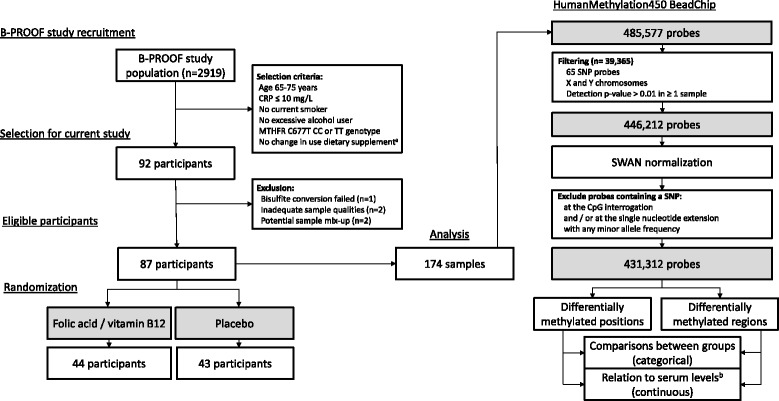
Flow diagram for the selection of participants and the analysis of samples. ^a^Self-reported change in use of dietary supplements containing folic acid or vitamin B_12_. ^b^Serum levels of folate or vitamin B_12_ and plasma levels of homocysteine. Abbreviations: *CRP* C-reactive protein, *MTHFR* methylenetetrahydrofolate reductase, *SNP* single nucleotide polymorphism, *SWAN* subset-quantile within array normalization

**Fig. 2:**
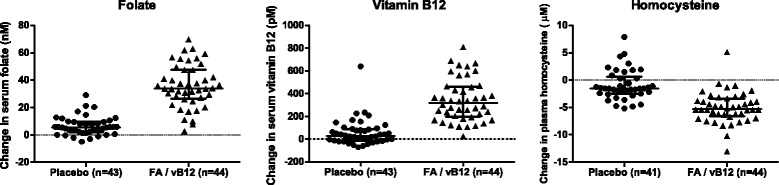
Changes in serum levels of folate, vitamin B_12_, and plasma levels of homocysteine. Individual changes in serum levels of folate and vitamin B_12_ and plasma levels of homocysteine after the 2-year intervention with folic acid and vitamin B_12_ or placebo. *Horizontal lines* represent median ± interquartile ranges

As shown in Fig. 1, 44 participants were randomized to the intervention with folic acid and vitamin B_12_, while 43 participants were assigned to the placebo group. Baseline characteristics of the participants are presented in Table 1. Inherent to the selection procedure for our study, participants with the methylenetetrahydrofolate reductase (*MTHFR*) (C677T) CC and TT genotypes were equally distributed among the groups. Age, body mass index, and serum levels of folate, vitamin B_12_, and plasma homocysteine at baseline did not differ between the two groups. The 2-year intervention with folic acid and vitamin B_12_ resulted in increased serum levels of both B-vitamins (folate median change 34.0 nmol/L; vitamin B_12_ median change 319 pmol/L) as well as decreased levels of homocysteine (median change −5.3 μmol/L). The changes in these levels were statistically significantly different from the ones observed in the placebo group (median change for folate 5.4 nmol/L, vitamin B_12_ 30.4 pmol/L, and homocysteine −1.5 μmol/L, for all parameters *p* < 0.0001). Individual changes in levels of serum folate, vitamin B_12_, and plasma homocysteine are presented in Fig. 2.

**Table 1:** Characteristics of the participants at baseline and after 2 years of intervention with either folic acid and vitamin B_12_ or placebo

	Folic acid and vitamin B_12_ (*n* = 44)	Placebo (*n* = 43)
Baseline characteristics		
Age at baseline (years)	70.8 ± 2.9	71.1 ± 3.0
Women (%)	25 (57 %)	22 (51 %)
Body mass index (kg/m^2^)	27.2 (24.4–29.7)	28.7 (25.7–31.2)
Smoking (%)^a^		
Former	25 (57 %)	31 (72 %)
Never	19 (43 %)	12 (28 %)
MTHFR C677T genotype (%)^b^		
CC	22 (50 %)	20 (47 %)
TT	22 (50 %)	23 (54 %)
Biochemical analyses		
Serum folate levels (nmol/L)		
Baseline	16.2 (13.1–24.4)	17.3 (14.1–21.5)
After intervention	52.3 (45.1–67.6)	23.1 (18.3–26.9)
Change	34.0 (26.5–47.6)	5.4 (2.0–9.6)
Serum vitamin B_12_ levels (pmol/L)		
Baseline	279 (232–358)	300 (204–365)
After intervention	595 (467–814)	325 (230–449)
Change	319 (197–462)	30.4 (−12.6–89.5)
Plasma homocysteine levels (μmol/L)		
Baseline	14.7 (13.1–16.3)	14.9 (13.1–16.3)
After intervention	9.6 (8.5–11.1)	13.7 (11.8–16.6)
Change	−5.3 (−6.7 to −3.4)	−1.5 (−2.4–0.6)

Data are presented as median (interquartile ranges) or numbers (%). ^a^Current smokers were not selected for the study population. ^b^Single nucleotide polymorphism: rs1801133, participants with the CT genotype were not selected for the current study

### Leukocyte proportions

In order to assess whether the mean leukocyte composition changed as a result of the intervention or duration of the study, leukocyte proportions were estimated based on DNA methylation data. The composition of the leukocyte fraction was similar for participants receiving folic acid and vitamin B_12_ or placebo and for both time points (Additional file 1: Figure S1). The Pearson correlation coefficient for estimated versus measured cell proportions in all follow-up samples (*n* = 85, 2 missing samples) was 0.66 for monocytes, 0.45 for lymphocytes (CD4+ and CD8+ T-lymphocytes, natural killer cells, and B-lymphocytes), and 0.46 for granulocytes (eosinophils, basophils, and neutrophils). No significant differences in measured or estimated cell proportions between the placebo and intervention group were measured at follow-up.

### Differentially methylated positions

**Fig. 3:**
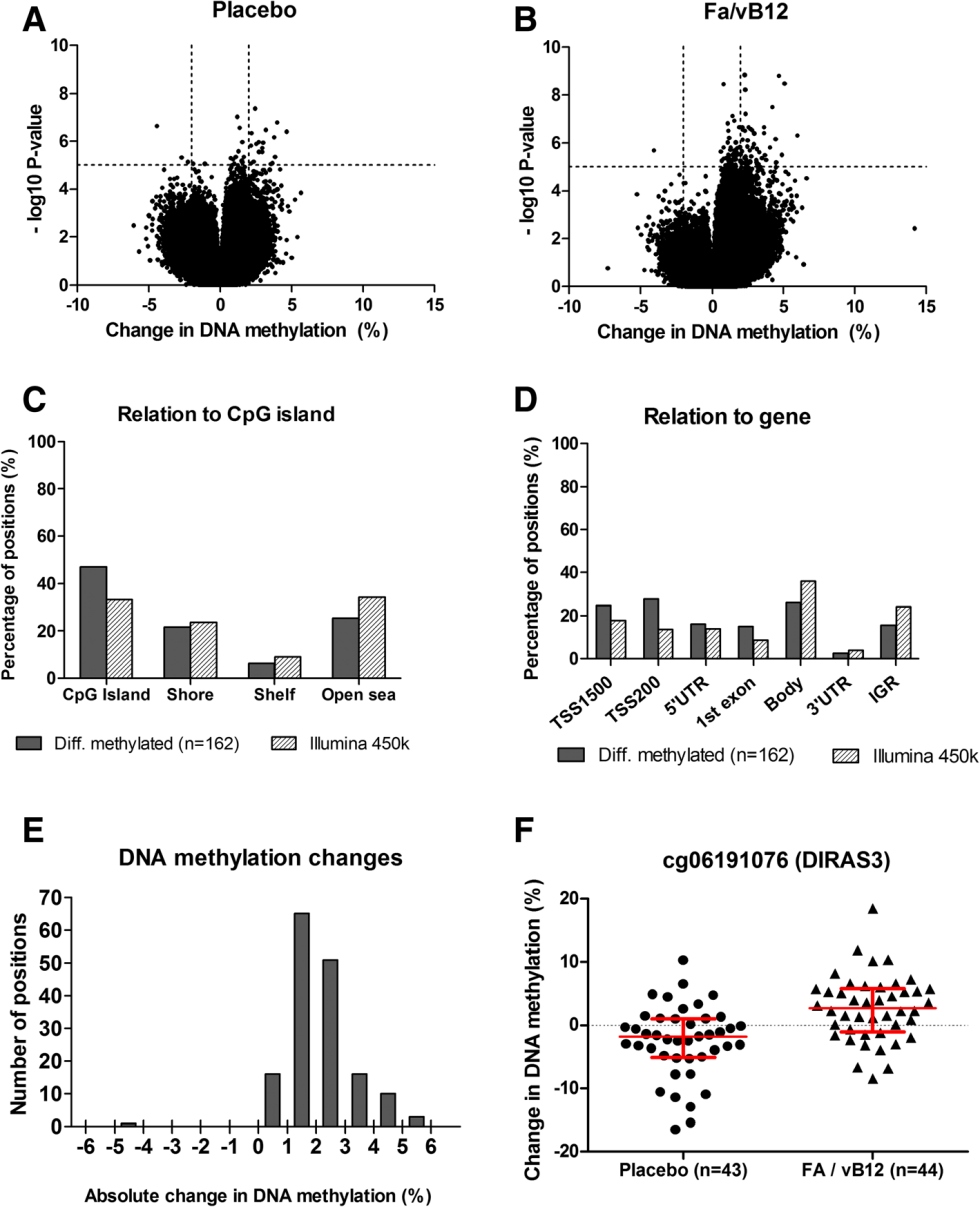
Differentially methylated positions after the intervention with folic acid and vitamin B_12_. Volcano plots show the statistical significance versus the changes in DNA methylation after the intervention with (**a**) the placebo or (**b**) folic acid and vitamin B_12_. *Dashed lines* represent 2 % methylation changes and a *p* value of 1.0E-05. Features of the positions (*n* = 162) that were differentially methylated after the intervention with folic acid and vitamin B_12_ are presented in (**c**) percentages of positions expressed per relationship to CpG islands for the differentially methylated positions (*n* = 162) as well as for all considered positions on the Infinium HumanMethylation450 Beadchip (*n* = 431,312), and (**d**) percentage of positions expressed per relationship to the nearest gene(s). **e** Number of positions according to the presented categories for absolute changes in DNA methylation for the 162 positions that were differentially methylated after the intervention with folic acid and vitamin B_12_. **f** Individual changes in DNA methylation for probe cg06191076 located within *DIRAS3. Horizontal lines* represent median ± interquartile ranges of DNA methylation, which is expressed as a beta value (0–100 %). Abbreviations: *TSS200* 200 base pairs around the transcription start site, *TSS1500* 1500 base pairs around the transcription start site, *3′UTR* 3′ untranslated region, *5′UTR* 5′ untranslated region, *IGR* intergenic region

At baseline, there were no statistically significant differences in methylation between the placebo and the intervention group. After intervention with folic acid and vitamin B_12_, 162 positions (versus 14 in the placebo group) of the 431,312 positions were differentially methylated as compared to baseline (Benjamini-Hochberg (BH)-adjusted *p* value <0.05). Volcano plots showing the DNA methylation changes versus the statistical significance are presented in Fig. 3a, b. An overview of the top-100 of differentially methylated positions with corresponding *p* values and methylation changes is presented in Additional file 2: Table S1. Mean changes in DNA methylation, as a result of the intervention, for these differentially methylated positions ranged from −4 to 5 % (Fig. 3e). Overall, positions within the CpG islands and around the transcription start sites were overrepresented among the positions that were differentially methylated after the intervention with folic acid and vitamin B_12_ (Fig. 3c, d).

### Differentially methylated positions that differ between groups

Comparisons between the DNA methylation changes in the participants receiving folic acid and vitamin B_12_ versus participants receiving the placebo showed one differentially methylated position with a BH-adjusted *p* value <0.05. This position (cg19380919) is located within a non-CpG dense region (open sea) on chromosome 2. Further exploration of the top-20 of differentially methylated positions showed that most pronounced differences (>5 %) were found for two probes on chromosome 4 and 1. For one probe (cg01129246) within the body of *GRXCR1*, mean DNA methylation increased with 2.6 % after the intervention with folic acid and vitamin B_12_, whereas it decreased with 4.1 % in the placebo group. Furthermore, DNA methylation changes differed for cg06191076 within the promoter region of the *DIRAS3* gene (also known as *ARHI*), a suggested tumor suppressor gene and member of the ras superfamily (Table 2). For this position, mean DNA methylation increased after intervention with folic acid and vitamin B_12_ with 2.7 %, whereas it decreased with 2.5 % in the placebo group (Fig. 3f). Stratification for MTHFR genotype did only reveal 2 and 3 (TT and CC, respectively) positions with differential methylation after the intervention with folic acid and vitamin B_12_, indicating that the power for stratified analyses may be insufficient as a consequence of the limited sample sizes in the subgroups.

**Table 2:** The top-20 of differentially methylated positions representing changes in DNA methylation that differ between participants receiving folic acid and vitamin B_12_ or placebo

						Mean change in methylation (%)	
CpG	Chr	Gene	Gene feature	*p* value	Adjusted *p* value	Folic acid and vitamin B_12_	Placebo
cg19380919	2	–	–	9.9E-08	0.0426	1.0	−1.4
cg05308617	3	ARMC8	TSS200	8.4E-07	0.1810	0.6	−0.6
cg06016645	10	NODAL	3′UTR	1.6E-06	0.2323	1.5	−0.8
cg11567849	6	REV3L	Body	2.7E-06	0.2495	0.8	−1.0
cg10367137	10	–	–	3.8E-06	0.2495	0.7	−1.1
cg18514573	6	–	–	4.0E-06	0.2495	0.6	−0.6
cg03330920	16	IFT140	Body	4.6E-06	0.2495	1.9	−2.1
cg01129246	4	GRXCR1	Body	4.9E-06	0.2495	2.6	−4.1
cg16895973	11	TOLLIP	Body	6.0E-06	0.2495	1.3	−2.6
cg09708616	6	–	–	6.3E-06	0.2495	1.4	−1.3
cg04530976	6	ZBTB9	Body	6.4E-06	0.2495	0.8	−0.9
cg27610561	20	SLC2A10	TSS1500	8.2E-06	0.2665	1.0	−1.6
cg05119686	10	–	–	8.7E-06	0.2665	1.5	−1.1
cg03761210	20	NECAB3;C20orf144	Body	8.7E-06	0.2665	0.3	−0.3
cg07292606	2	TEX261	Body	9.4E-06	0.2665	2.0	−0.9
cg27194173	10	ADK	Body	9.9E-06	0.2665	1.9	−0.9
cg06191076	1	DIRAS3	TSS200	1.3E-05	0.3192	2.7	−2.5
cg00066153	11	ACRV1	1stExon; 5′UTR	1.3E-05	0.3192	1.1	−0.9
cg20455570	7	OSBPL3	5′UTR	1.7E-05	0.3888	1.2	−1.0
cg24106824	17	MRPL12	3′UTR	2.0E-05	0.4105	1.1	−0.6

Mean changes in methylation are presented as beta-values ranging from 0–100 %. The adjusted *p* values for the comparisons between the two groups control the false discovery rate using the Benjamini-Hochberg procedure. Abbreviations: *Chr* chromosome, *TSS200* 200 base pairs around the transcription start site, *TSS1500* 1500 base pairs around the transcription start site, *3′UTR* 3′ untranslated region, *5′UTR* 5′ untranslated region

### Differentially methylated regions that differ between groups

**Fig. 4:**
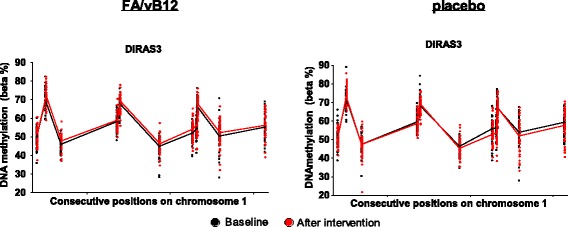
The differentially methylated region within *DIRAS3*. Individual (*dots*) and median (*lines*) DNA methylation values for the positions within the identified differentially methylated regions (DMRs) for *DIRAS3* (chromosome 1) before (*black*) and after (*red*) the intervention with folic acid and vitamin B_12_ (*n* = 44) or placebo (*n* = 43). The DMRs were identified using the DMRcate package [81]. Coordinates for the human genome are based on h19/GRCh37

Identification and characterization of differentially methylated regions (DMRs), consisting of multiple consecutive positions, showed that 6 regions differed for the participants receiving folic acid and vitamin B_12_ versus placebo (BH-adjusted *p* value <0.05) (Table 3). Again, for a DMR of 11 positions within *DIRAS3* the most pronounced difference (maximal 5.2 %) in DNA methylation changes was found (Table 3 and Fig. 4). For this DMR, the mean percentage of DNA methylation increased after intervention with folic acid and vitamin B_12_ (1.5 %), but decreased in the placebo group (−0.9 %). A similar pattern was found for *NODAL* (nodal growth differentiation factor) on chromosome 10, for which 2 consecutive positions located within the 3′ untranslated region (3′UTR) were identified. After intervention with folic acid and vitamin B_12_, the mean methylation for these positions increased with 1.2 %, whereas in the placebo group a modest decrease of −0.4 % was found.

**Table 3:** Differentially methylated regions with DNA methylation changes that differ between participants receiving folic acid and vitamin B_12_ or placebo

Gene	Gene feature	Chr	hg19 coordinates	Probes	Minimal *p* value	Mean *p* value	Maximal difference in methylation change (%)^a^
DIRAS3	5′UTR,1stExon,TSS200	1	68516080-68516627	11	0.0095	0.0175	5.2
–	–	6	85478576-85478807	2	0.0155	0.0210	3.6
NODAL	3′UTR	10	72191686-72191804	2	0.0198	0.0231	2.3
ARMC8	TSS1500,TSS200	3	137905836-137906122	7	0.0361	0.0400	1.3
PRDM16	Body	1	3130485-3130550	3	0.0361	0.0361	2.8
GNA12	Body	7	2801424-2801732	3	0.0387	0.0437	−2.4

The DMRs were identified using the DMRcate package available through Bioconductor. The *p* values considered the false discovery rate and were adjusted using the Benjamini-Hochberg procedure. The minimal and mean *p* value as well as the maximal difference in DNA methylation change were calculated based on the indicated number of probes for each region. ^a^The maximal difference was calculated for the DNA methylation change after intervention with folic acid and vitamin B_12_ versus placebo. Abbreviations: *Chr* chromosome, *DMR* differentially methylated region, *h19* coordinates for the human genome based on the Genome Reference Consortium Human Build 37 (hg19/GRCh37), *TSS200* 200 base pairs around the transcription start site, *TSS1500* 1500 base pairs around the transcription start site, *3′UTR* 3′ untranslated region, *5′UTR* 5′ untranslated region

### Relation with serum or plasma levels of B-vitamins and homocysteine

**Fig. 5:**
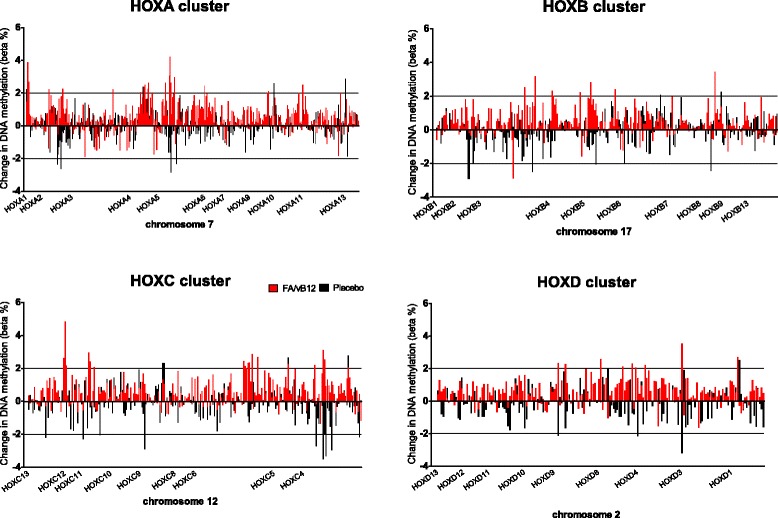
The effect of folic acid and vitamin B_12_ on DNA methylation of *HOX* genes. Mean DNA methylation changes after a 2-year intervention with folic acid and vitamin B_12_ (*red*, *n* = 44) or placebo (*black*, *n* = 43) for the 1052 positions located within one of the 39 *HOX* (homeobox) genes located on cluster A (chromosome 7), cluster B (chromosome 17), cluster C (chromosome 12), or cluster D (chromosome 2). Only positions annotated to one of these *HOX* genes were depicted; intergenic regions were not included in this figure. *Bars* are superimposed, meaning that *red* (folic acid and vitamin B_12_) and *black* (placebo) *bars* are presented together, whenever applicable behind each other, and both reflect the actual values on the *y*-axis

Changes in serum levels of folate or vitamin B_12_ and plasma homocysteine after the 2-year intervention period were fairly subjected to interindividual variation resulting in an “overlap” between the intervention and placebo group (Fig. 2). In addition to the comparisons for the DNA methylation changes between these two groups, we therefore explored the relation between DNA methylation and serum folate, vitamin B_12_ or plasma homocysteine levels in a continuous manner. Applying the conservative threshold for significance of BH-adjusted *p* values <0.05 revealed no statistically significant positions related to folate levels. Exploration, however, of the top-35 positions for which DNA methylation tends to be related to serum folate levels showed a probe (cg24973150, *p* = 5.15E-05) located within a CpG island in the promoter region (TSS200) of the *HOXB7* gene, which has been recently associated with the risk of neural tube defects [31]. Most (32 of the top-35 positions) of the observed positions were positively related to serum levels of folate, indicating that DNA methylation increased with higher levels of serum folate. In addition to the identification of individual positions, we subsequently identified 173 DMRs that were related to folate levels. One of the most prominent regions, consisting of 22 probes and a mean BH-adjusted *p* value of 0.001, belongs to the promoter region of *HOXA4*, another member of the homeobox family. An overview of DNA methylation changes in *HOXB7*, *HOXA4*, and the other *HOX* genes in one of the four *HOX* gene clusters is presented in Fig. 5, showing that mean DNA methylation for the majority of these *HOX* genes tends to be increased after intervention with folic acid and vitamin B_12_, whereas it mostly remained stable or decreased in the placebo group.

**Fig. 6:**
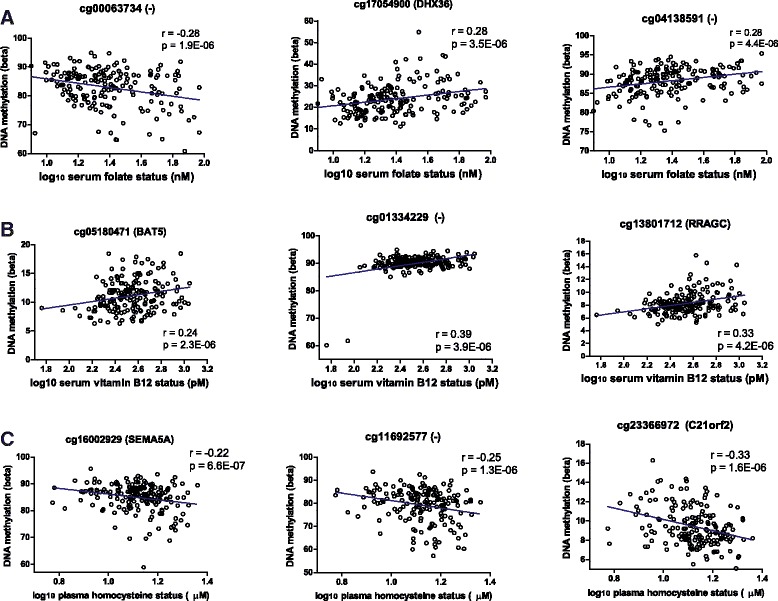
The relation between serum levels of folate, vitamin B_12_ and plasma homocysteine and DNA methylation. The top-3 of the positions (based on lowest *p* values derived from the limma regression analyses) related to **a** serum folate levels, **b** serum vitamin B_12_ levels, and **c** plasma homocysteine levels. Linear regression lines are presented in blue and the Pearson correlation coefficient (*r*) is presented. *P* values refer to non-adjusted *p* values from the limma regression analyses. All samples (*n* = 174) were included in the analyses, except for plasma homocysteine for which two samples were excluded because of missing values

Likewise, for plasma levels of homocysteine, 11 DMRs were identified. As expected, an inverse association between DNA methylation and plasma levels of homocysteine was found for most (32 of the top-35 positions) of the positions related to homocysteine levels. In comparison to folate and homocysteine, most pronounced relations with DNA methylation were, however, found for serum levels of vitamin B_12_. In total, 425 regions were identified for which DNA methylation was related to serum vitamin B_12_ levels (BH-adjusted *p* value <0.05). An overview of the top-3 most statistically significant positions related to folate, vitamin B_12_, or homocysteine levels is presented in Fig. 6. A detailed list with differentially methylated positions and regions related to either folate, vitamin B_12_, or homocysteine levels is included in Additional file 3: Table S2.

## Discussion

The current study shows that long-term supplementation with folic acid and vitamin B_12_ resulted in DNA methylation changes in leukocytes of older persons. We identified several differentially methylated positions as well as regions for which the change in DNA methylation differed between the participants receiving folic acid and vitamin B_12_ versus placebo. Furthermore, the DNA methylation levels of several genomic loci were found to correlate to serum levels of either folate, vitamin B_12_, or plasma homocysteine. Most prominent DNA methylation patterns associated with supplemental intake or status of these B-vitamins seemed to be related to developmental processes as well as carcinogenesis.

Based on comparisons between the two groups (folic acid and vitamin B_12_ versus placebo), *DIRAS3* was identified as a gene of interest with a DMR consisting of 11 consecutive positions. *DIRAS3*, also known as *ARHI*, is a maternally imprinted member of the ras superfamily and is considered to be a tumor suppressor gene [32]. Downregulation of *DIRAS3* expression has been described for several forms of cancer and has been specifically associated with progression and invasive behavior of neoplastic cells [33–35]. To what extent transcriptional downregulation of *DIRAS3* occurs via epigenetic mechanisms is not exactly known, although previous work indicated that regulation by miRNAs, chromatin remodeling as well as promoter hypermethylation may be responsible for this effect [35–37].

Another gene identified based on its DMR responsive to the intervention with folic acid and vitamin B_12_ is the nodal growth differentiation factor (*NODAL*), a member of the transforming growth factor-β (*TGF-beta*) superfamily. Like *DIRAS3*, *NODAL* has been recognized for its role in cancer progression, with abundant expression of *NODAL* associated with cellular migration, invasion, and metastatic behavior [38–41].

Noteworthy, *NODAL* is not only known for its role in cancer progression as it has been originally identified as a morphogen and regulator of mesoderm formation and organization of axial structures during early-stage embryogenesis [42, 43]. Upregulation of *NODAL* expression as a consequence of tobacco or nicotine exposure in differentiating human embryonic stem cells (hESCs) suggests that the *NODAL* signaling routes are vulnerable to environmental exposures or stimuli during embryonic development [44]. Interestingly, it has been recently demonstrated that expression of *NODAL* is regulated via an epigenetic regulatory element for which dynamic changes in DNA methylation were described throughout embryonic development [45]. Although this epigenetic regulatory element was found upstream of the *NODAL* gene, whereas our small DMR was located in the 3′UTR, these results support our findings that *NODAL* may be sensitive to endogenous or environmental exposures.

Besides *NODAL*, also positions or regions within other developmental genes have been identified as potentially responsive to the intervention with folic acid and vitamin B_12_ or were related to serum folate levels in our study. The homeobox B7 (*HOXB7*) gene was identified for its relation with serum folate levels of the participants in the current study. *HOXB7* is a member of the homeobox family [46] and occurs in a cluster with other homeobox B genes. For *HOXA4*, a DMR was related to serum folate levels. The highly conserved *HOX* genes are considered important transcriptional regulators during embryonic development, where they are mainly responsible for vertebral axial patterning [47, 48].

Epigenetic control of *HOX* genes in developmental processes related to health and disease has been described previously [47, 49]. Hypomethylation of *HOXB7* has been recently recognized as a risk factor for neural tube defects in a case-control study [31]. It is firmly established that periconceptional folic acid supplementation decreases the risk of neural tube defects in the offspring [50, 51]. To what extent *HOX* or other developmental genes are key targets for the prevention of neural tube defects through folic acid supplementation has not been described extensively so far [52]. The clinical relevance of our findings with respect to mechanisms underlying prevention of neural tube defects, however, is questionable since we found modest increases (~2–5 %) in DNA methylation of the *NODAL* or *HOX* genes after supplementation in our elderly population. Case-control studies have previously shown that DNA methylation of several *HOX* genes differed up to 29 % between children with myelomeningocele (a form of spina bidifa) and healthy controls [31]. To what extent epigenetic control of developmental genes during fetal development [1] can be translated into an aging population, and vice versa, is not clear. Thus, our careful and explorative hypotheses about the effects of folic acid and vitamin B_12_ on epigenetic processes in relation to programming of (early) developmental genes warrant further investigation and confirmation in future research with a specific focus on the suggested interaction between genetic, biological, and nutritional factors [53].

As stated before, aberrant DNA methylation or expression of several *HOX* genes, *NODAL* and *DIRAS3* have been implicated in the etiology of cancer [54, 55]. Members of the homeobox family as well as *NODAL* are specifically associated with cancer progression and metastatic behavior [56–59]. Reactivation of embryonic pathways in cancer cells may contribute to uncontrolled differentiation, proliferation, invasion, and metastatic behavior. To what extent the suggested increases in DNA methylation of tumor-related genes such as *DIRAS3*, *NODAL*, *HOXB7*, *HOXA4*, and other *HOX* genes may contribute to the pathogenesis of cancer cannot be concluded from the current study. Theoretically, our findings, however, support the hypothesis that folic acid and possibly other B-vitamins specifically increase cancer risk if preclinical neoplastic lesions already exist, pointing toward an effect on progression rather than development of cancer [27, 29]. Our findings may also explain the explorative results of the entire B-vitamins for the PRevention Of Osteoporotic Fractures (B-PROOF) study, where a slightly increased cancer risk was reported after intervention with folic acid and vitamin B_12_ as compared to the placebo group (hazard ration 1.56; 95 % confidence interval 1.04-2.30) [60]. Taken altogether, our data suggest that supplementation with folic acid and vitamin B_12_, and consequential changes in serum levels of the corresponding B-vitamins or plasma homocysteine, in older subjects is associated with changes in DNA methylation of several genes implicated in normal developmental processes, which may be reactivated or deregulated during carcinogenesis. It should be noted that the observed effects on DNA methylation were marginal and that biological and functional consequences with respect to gene expression cannot be validated with the current study. On the other hand, previous studies from our groups and others have already shown that nutritional or environmental exposures often result in small, but biologically meaningful, effects on DNA methylation in comparison to the tremendously deviating methylation patterns observed in complex diseases such as cancer [61–63].

Potential limitations of our study include the relatively heterogeneous study population with respect to characteristics that may have occurred or changed during the intervention period, such as changes in dietary habits, use of drugs, and presentation of diseases. It should be noted, however, that these characteristics represent and reflect the general elderly population, which is therefore an advantage with respect to the generalizability of our findings. For disease-related findings, it is questionable whether our results observed in leukocytes also reflect the situation in the tissue of interest, since DNA methylation patterns are highly tissue specific [64–66]. Finally, all participants in our study received 15 μg of vitamin D_3_ per day in order to ensure a normal vitamin D status. Therefore, we cannot exclude the possibility that some DNA methylation changes may be predominantly attributable to vitamin D rather than folic acid and vitamin B_12_ [67]. However, since all participants, both from the intervention group and the placebo group, received vitamin D in their study tablets (also containing the placebo or folic acid/vitamin B_12_), this did not hinder our main analyses dedicated to comparisons between these two groups. Although, the results from our study were fairly consistent with other studies and current insights, strict replication of our findings was not feasible so far given the unique design of our long-term intervention study with folic acid as well as vitamin B_12_ among elderly subjects.

Strengths of our study include the analyses of genome-wide DNA methylation both before and after long-term supplementation, which allow comparisons within individuals over time. Furthermore, our population consisted of older subjects with mildly elevated homocysteine levels. Elevated homocysteine levels, which are common among elderly subjects in The Netherlands [68], may point toward inadequate levels or deficiencies of folate and vitamin B_12_ [69]. Therefore, especially, this population may have benefited from the prolonged supplementation with these B-vitamins and thus enabled detection of changes in DNA methylation patterns.

## Conclusions

Long-term supplementation with folic acid and vitamin B_12_ in elderly subjects with mildly elevated homocysteine levels resulted in changes in DNA methylation of several genes implicated in normal developmental processes and carcinogenesis. These findings may provide unique leads for further research unraveling the mechanisms underlying the effects of B-vitamins on health and disease during the life cycle.

## Methods

### Design and recruitment

The current study was part of a double-blind, randomized, and placebo-controlled trial on the effects of supplemental intake of folic acid and vitamin B_12_ on fracture incidence (B-PROOF) in The Netherlands [60, 70]. Design of the B-PROOF study and recruitment of the participants have been described in detail previously [70]. Briefly, men and women, aged 65 years and older, with an elevated homocysteine level (12–50 μmol/L) were eligible to participate in this study. Between September 2008 and March 2011, over 69,000 subjects were screened and 2919 participants were finally enrolled in the B-PROOF study (Fig. 1). All participants provided written informed consent and the study protocol was approved by the institutional Medical Ethics Committee. Participants were randomly assigned to take 400 μg folic acid and 500 μg vitamin B_12_ (Orthica, Almere, The Netherlands) per day or a placebo (Orthica, Almere, The Netherlands) during an intervention period of 2 years. Moreover, all participants received a daily dose of 15 μg vitamin D_3_, in the same tablet as the placebo or B-vitamins, to ensure a normal vitamin D status.

For the current study, a subgroup of participants was selected from the B-PROOF study cohort. From the 331 participants, who were among the first who completed the intervention period of 2 years, 92 participants were selected for DNA methylation analyses. In order to obtain a relatively homogenous study population, our predefined selection criteria included: age between 65 and 75 years and no reported changes in use of dietary supplements containing folic acid or vitamin B_12_ during the study. Levels of the C-reactive protein (CRP) at baseline should be ≤10 mg/L, because elevated levels of CRP as a consequence of inflammation may indicate aberrant leukocyte counts. Furthermore, participants who were current smokers, or excessive alcohol users (at least once a week ≥6 glasses of alcohol or 5–7 days/week ≥4 glasses of alcohol) [71] at baseline were not selected for the current study. All participants had elevated homocysteine levels, which is in line with inclusion criteria of the B-PROOF study. Given the essential role of MTHFR in folate-mediated one-carbon metabolism, the functional C677T single nucleotide polymorphism (SNP) of the *MTHFR* gene was considered as well for the selection procedure. Participants with the *MTHFR* (C677T) CC (*n* = 46) and TT (*n* = 46) genotypes were equally and exclusively represented in the current study. The *MTHFR* SNP was determined using the Illumina Omni-express array (Illumina Inc., San Diego, CA, USA).

### Sample collection and biochemical analyses

At baseline and after 2 years of intervention, blood samples were collected from all 92 participants. Serum levels of folate and vitamin B_12_ were determined using electrochemiluminescence immunoassays (Elecsys 2010, Roche, Almere, The Netherlands). Depending on the study center, plasma levels of homocysteine were measured using the Architect i2000 RS analyzer (VU University Medical Center, Amsterdam), HPLC (Wageningen University, Wageningen), or LC-MS/MS method (Erasmus MC, Rotterdam). According to a cross-calibration, outcomes of the three centers did not differ significantly [60].

### Genome-wide DNA methylation analysis

DNA was isolated from buffy coats and 500 ng genomic DNA was bisulfite converted using the EZ DNA Methylation Kit according to the manufacturer instructions (Zymo Research, Freiburg, Germany). Bisulfite converted DNA was used for the genome-wide DNA methylation analyses. Genome-wide DNA methylation was determined in a total of 184 samples from 92 participants using the Infinium HumanMethylation450 BeadChip (Illumina). All samples were processed and analyzed at the Genetic laboratory of Internal Medicine of the Erasmus MC, Rotterdam, The Netherlands. Initial quality control of the samples revealed that one participant had to be excluded because bisulfite conversion for the baseline sample failed. Furthermore, two participants were excluded since their baseline samples showed (1) an average intensity signal in the methylated or unmethylated channel below our predefined threshold of 1966 and (2) a percentage of probes with a call rate <97.5 %. Clustering based on the 65 known SNP probes on the BeadChip as well as multidimensional scaling stratified for gender, revealed two potential sample mix-ups. The corresponding participants of these samples were excluded resulting in samples from 87 participants available for final analysis (Fig. 1).

Raw idat.files were subsequently used for analysis in the R statistical environment (R 3.1.2) through the minfi package (version 1.12.0) available from Bioconductor [72]. Preprocessing of the data included filtering of 65 control probes containing known SNPs and probes annotated to the X and Y chromosome. Probes with a detection *p* value of >0.01 in at least one sample were filtered, as these probes did not surpass the background signal. Finally, probes containing an SNP (based on the dbSNP version 137) [73] at the CpG interrogation and/or the single nucleotide extension were excluded, resulting in 431,312 probes included in the analyses (Fig. 1).

Filtered data were normalized using the SWAN normalization procedure [74] available through the minfi package [72]. The datasets were annotated based on the ilmn12.hg19 annotation [75]. The M-values, which are the log2 ratios of the methylated versus the unmethylated probe intensities, were used for statistical testing as recommended by Du et al. [76]. For visualization purposes, methylation status of the individual probes was expressed as a β-value. These β-values range from 0 or 0 % (fully unmethylated) to 1 or 100 % (fully methylated). Phenotypic data and idat-files from the genome-wide DNA methylation analysis are available through the NCBI’s Gene Expression Omnibus (GEO) repository [77] with accession number GSE74548.

### Differentially methylated positions

Initially, differentially methylated positions, representing changes in DNA methylation over time, were identified in both the placebo as well as the intervention group using a multi-level linear regression analysis within the limma package [78, 79]. For these analyses, subjects were treated as random effects using the “duplicateCorrelation” function in order to conduct the comparisons in a pairwise manner [78, 80].

Differences in changes in DNA methylation between the placebo and intervention group were identified using this multi-level linear regression analysis as well [78, 80]. The contrast ((Placebo_followup-Placebo_baseline)-(Intervention_followup-Intervention_baseline)) allowed the comparison between DNA methylation changes in the two groups.

Besides the comparisons between the two groups, differentially methylated positions were identified as a function of serum folate, vitamin B_12,_ or plasma homocysteine levels using the dmpFinder function for continuous phenotypes (available from the minfi package [72]). For these analyses, log10-transformed serum levels of folate, vitamin B_12_ levels, or plasma homocysteine at baseline and after intervention were considered as continuous variables in the linear regression analyses (*n* = 174).

### Differentially methylated regions

Identification of differentially methylated regions (DMRs) was conducted using the DMRcate package available through Bioconductor [81]. DMRs were defined as regions with a maximal distance of 1000 nucleotides between consecutive probes [81]. Identified DMRs consisting of a single probe were excluded for further analyses. Unless otherwise stated, we have taken into account the issue of multiple testing for all analyses by considering *p* values which are adjusted for the false discovery rate (FDR) by using the Benjamini-Hochberg (BH) procedure [82].

### Statistical considerations

Samples with a heterogeneous cell population are a major issue for epigenomic analyses, since DNA methylation shows a pronounced cell-type specific pattern [65]. In order to determine whether the leukocyte fractions differed between the groups or baseline and follow-up samples, we predicted the leukocyte composition using the Houseman method [83, 84] implemented in minfi [72]. For a subgroup of participants (*n* = 85, follow-up samples only), automated cell counts (lymphocytes, monocytes and basophil, eosinophil and neutrophil granulocytes) were available. Pearson correlation coefficients were calculated for the predicted and measured counts of these specific leukocyte fractions. The statistical analyses were adjusted for the different cell fractions by inclusion of predicted percentages of CD4+ and CD8+ T-lymphocytes, B-lymphocytes, natural killer cells, monocytes, and granulocytes as covariates in the linear regression models. Also, the plates used for the bisulfite conversion (four in total) were included as covariates in the models.

Since numerous demographic and biochemical variables were not normally distributed, these data were summarized as median and interquartile ranges (IQR) or numbers and percentages. The Mann-Whitney *U* test was used to compare characteristics and changes in serum levels of folate, vitamin B_12_, or plasma homocysteine between the intervention group and the placebo group. The Statistical package for Social Sciences (SPSS version 22) was, unless otherwise stated, used for all the statistical analyses regarding the descriptive and biochemical parameters.

## Additional files

Additional file 1: Figure S1. The mean proportion of the leukocyte fractions in the buffy coat samples collected before and after intervention with folic acid and vitamin B12 (n=44) or placebo (n=43). Estimates of the leukocyte fractions were based on the Houseman method. Additional file 2: Table S1. Top-100 positions that are differentially methylated after intervention with folic acid and vitamin B12. Overview of the top-100 positions that are differentially methylated (Benjamini-Hochberg adjusted p-value <0.05) after intervention with folic acid and vitamin B12 (FA/vB12). Changes for DNA methylation in the intervention group as well as the placebo group are presented as % beta-values and can range from 0-100 %. Additional file 3: Table S2a and S2b. Differentially methylated positions and regions related to serum levels of folate, vitamin B12 of plasma homocysteine. Overview of the top-10 of a) differentially methylated positions as well as b) regions related to serum levels of folate, vitamin B12 of plasma homocysteine.

## Abbreviations

3′UTR: 3′ untranslated region
5′UTR: 5′ untranslated region
BH: Benjamini-Hochberg
B-PROOF: B-vitamins for the prevention of osteoporotic fractures
CpG: cytosine-phosphate-guanine
CRP: C-reactive protein
DMR: differentially methylated region
FA/vB12: folic acid and vitamin B_12_
FDR: false discovery rate
hESC: human embryonic stem cell
HPLC: high-performance liquid chromatography
IQR: interquartile range
LC-MS/MS: liquid chromatography-tandem mass spectrometry
MTHFR: methylenetetrahydrofolate reductase
SNP: single nucleotide polymorphism
SWAN: subset-quantile within array normalization
TSS: transcription start site
