# Supplementary figures and images for: The effects of long-term daily folic acid and vitamin B12 supplementation on genome-wide DNA methylation in elderly subjects
Source: Clin Epigenetics. 2015 Nov 14;7:121. doi: 10.1186/s13148-015-0154-5 (PMC4644301; doi:10.1186/s13148-015-0154-5)

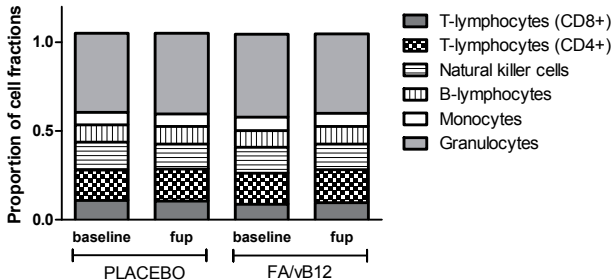

Supplement: Additional file 1: Figure S1. — The mean proportion of the leukocyte fractions in the buffy coat samples collected before and after intervention with folic acid and vitamin B12 (n=44) or placebo (n=43). Estimates of the leukocyte fractions were based on the Houseman method. [file 13148_2015_154_MOESM1_ESM.pdf]
