# Supplementary material for: The effects of long-term daily folic acid and vitamin B12 supplementation on genome-wide DNA methylation in elderly subjects
Source: Clin Epigenetics. 2015 Nov 14;7:121. doi: 10.1186/s13148-015-0154-5 (PMC4644301; doi:10.1186/s13148-015-0154-5)
Supplement: Additional file 2: Table S1. — Top-100 positions that are differentially methylated after intervention with folic acid and vitamin B12. Overview of the top-100 positions that are differentially methylated (Benjamini-Hochberg adjusted p-value <0.05) after intervention with folic acid and vitamin B12 (FA/vB12). Changes for DNA methylation in the intervention group as well as the placebo group are presented as % beta-values and can range from 0-100 %. [file 13148_2015_154_MOESM2_ESM.pdf]

Supplementary Table S1: Top-100 positions that are differentially methylated after intervention with folic acid and vitamin B12. Overview of the top-100 positions that are differentially methylated (Benjamini-Hochberg-adjusted p-value <0.05) after intervention with folic acid and vitamin B12 (FA/vB12). Changes for DNA methylation in the intervention group as well as the placebo group are presented as % beta-values and can range from 0-100%.

Position	P-value	adj.P.Val	chr	Gene(s)	Methylation change for FA/vB12 (%)	Methylation change for placebo (%)
cg27023953	1.5E-09	0.0004	22	C22orf41	2.3	0.7
cg11588710	1.6E-09	0.0004	3	-	4.7	2.4
cg22561380	3.4E-09	0.0004	8	HOOK3	5.1	3.0
cg02360155	3.6E-09	0.0004	4	-	0.8	0.4
cg26087862	6.2E-09	0.0005	1	ENSA	2.3	1.7
cg26561082	3.2E-08	0.0023	10	DIP2C	4.3	4.0
cg16308101	6.2E-08	0.0038	1	SERBP1	2.4	1.0
cg18944135	7.6E-08	0.0041	10	-	1.4	0.0
cg10664184	1.2E-07	0.0057	19	DDA1	1.6	0.6
cg01781745	1.5E-07	0.0067	9	KLF9	1.1	-0.1
cg08494150	2.2E-07	0.0070	16	HS3ST4	2.6	2.0
cg04402803	2.2E-07	0.0070	12	BLOC1S1	3.7	1.3
cg21641186	2.2E-07	0.0070	3	UROC1	1.9	0.1
cg14749334	2.3E-07	0.0070	19	RAB11B	2.1	1.0
cg05055652	2.6E-07	0.0072	7	ATP6V1F	2.7	1.3
cg14458002	2.8E-07	0.0072	6	RUNX2	2.2	0.0
cg13089318	2.9E-07	0.0072	1	-	2.8	1.9
cg02070354	3.1E-07	0.0072	2	RQCD1;USP37	1.2	1.0
cg16429819	3.2E-07	0.0072	3	GPX1	2.3	0.9
cg05522145	3.9E-07	0.0084	7	-	3.6	1.5
cg16711165	4.1E-07	0.0084	11	TIMM8B;SDHD	3.0	2.4
cg17054900	5.0E-07	0.0098	3	DHX36	6.0	2.5
cg06880857	6.3E-07	0.0118	14	DICER1	1.2	0.1
cg00062070	7.0E-07	0.0125	15	C15orf41	4.5	-0.6
cg12631604	7.3E-07	0.0125	21	ATP5O	2.1	0.6
cg04138591	7.7E-07	0.0128	7	-	2.5	1.4
cg20488820	9.5E-07	0.0147	3	-	1.1	-0.1
cg26088104	9.5E-07	0.0147	5	C5orf34	1.7	0.8
cg13022888	1.2E-06	0.0172	14	SEL1L	2.9	2.4
cg10714940	1.4E-06	0.0196	3	GTF2E1;RABL3	2.5	2.2
cg24406775	1.5E-06	0.0196	11	SLC3A2	2.0	-0.2
cg17014947	1.5E-06	0.0196	15	LOC723972	2.9	1.9
cg04915044	1.6E-06	0.0196	2	POLR1A	4.1	2.5
cg01459962	1.6E-06	0.0196	16	CCDC101	0.8	0.3
cg03067660	1.6E-06	0.0196	6	KLC4;MRPL2	1.3	0.5
cg03414764	1.7E-06	0.0201	15	KIAA1199	1.8	0.8
cg00195828	1.8E-06	0.0206	8	MFHAS1	3.2	0.9
cg03986574	1.9E-06	0.0208	11	SF1	1.3	0.3
cg25627714	1.9E-06	0.0208	2	NEURL3	1.7	0.5
cg11755933	2.0E-06	0.0208	11	PITPNM1	1.3	0.5
cg12320164	2.0E-06	0.0208	17	LOC645638	-4.1	0.4
cg26985354	2.1E-06	0.0208	19	SFRS16	2.7	1.4
cg24477193	2.1E-06	0.0208	1	ALG14	1.6	0.1
cg22963133	2.2E-06	0.0217	5	ARL15	2.1	1.1
cg19565299	2.3E-06	0.0222	2	D2HGDH	0.6	0.4
cg11754259	2.5E-06	0.0228	11	ZBTB44	1.6	0.1
cg20699097	2.5E-06	0.0228	11	TIMM8B;SDHD	3.3	1.7
cg02009091	2.5E-06	0.0228	19	KLK13	1.7	-0.3
cg03133777	2.9E-06	0.0253	2	BBS5	2.0	-0.1
cg13168924	3.0E-06	0.0253	19	NDUFA7;RPS28	1.8	1.5
cg09566392	3.0E-06	0.0253	2	PQLC3	2.0	0.7
cg06601475	3.2E-06	0.0257	13	GPC5	1.2	-0.2
cg03651886	3.2E-06	0.0257	10	-	1.5	0.2
cg18487343	3.2E-06	0.0257	14	-	4.9	3.4
cg07240689	3.3E-06	0.0261	10	PDCD11	3.7	0.1
cg13962681	3.4E-06	0.0261	1	RPS6KC1	1.2	0.1
cg25420583	3.5E-06	0.0261	2	WBP1	1.2	0.8
cg21191822	3.5E-06	0.0261	6	ZBTB9	1.7	1.1

cg02674114	3.7E-06	0.0268	1	ERI3	2.8	1.6
cg10616672	3.8E-06	0.0270	12	C12orf35	2.4	0.3
cg07060117	3.9E-06	0.0273	16	CFDP1	1.6	0.5
cg03736944	4.3E-06	0.0298	16	RAB40C	2.3	1.2
cg25550279	4.4E-06	0.0300	7	-	3.5	0.8
cg17864619	4.5E-06	0.0300	19	FOSB	1.0	0.5
cg24612305	4.6E-06	0.0304	17	TOM1L2;LRRC48	0.8	0.2
cg13685680	4.8E-06	0.0312	12	FBRSL1	1.7	0.4
cg13529619	4.9E-06	0.0316	3	RAD18	1.5	1.0
cg23461432	5.1E-06	0.0316	11	OR8U8	1.3	-0.4
cg18443378	5.1E-06	0.0316	4	WDR17	2.4	0.0
cg10746622	5.2E-06	0.0318	19	PRSSL1	3.8	-0.1
cg17112695	5.2E-06	0.0318	12	AGAP2	1.6	0.4
cg19496566	5.3E-06	0.0319	19	GLTSCR2	3.1	1.6
cg16434365	5.5E-06	0.0320	16	-	2.0	-0.3
cg07428804	5.5E-06	0.0320	11	STX5	1.2	0.7
cg06639079	5.7E-06	0.0327	19	C19orf36;MOBKL2A	0.7	0.3
cg17188367	5.9E-06	0.0329	6	RNF5P1;AGPAT1	2.7	0.9
cg04020138	5.9E-06	0.0329	3	-	2.0	0.0
cg16101101	6.4E-06	0.0348	1	ILF2	2.7	2.5
cg01188822	6.5E-06	0.0348	6	YIPF3;POLR1C	1.2	0.9
cg01276222	6.5E-06	0.0348	5	CCNB1	3.1	1.1
cg24927841	6.6E-06	0.0348	8	-	4.5	-1.8
cg04166874	6.6E-06	0.0348	1	ERI3	2.6	1.0
cg08365438	7.0E-06	0.0352	10	-	2.2	0.9
cg14407075	7.0E-06	0.0352	12	LARP4	2.6	1.6
cg04214573	7.0E-06	0.0352	10	ACSL5	2.1	0.8
cg16349277	7.0E-06	0.0352	4	-	4.5	4.0
cg20977411	7.3E-06	0.0360	1	PMVK	1.4	0.9
cg09791459	7.4E-06	0.0360	16	GFOD2	2.3	1.6
cg05871614	7.5E-06	0.0365	22	NHP2L1	3.3	1.3
cg21581312	7.7E-06	0.0368	15	LOC723972	3.2	2.0
cg00999186	8.1E-06	0.0384	3	-	1.6	-0.3
cg22376739	8.3E-06	0.0391	17	TOM1L2;LRRC48	1.1	0.2
cg26953093	8.4E-06	0.0391	14	SIVA1	1.4	0.8
cg26496372	8.6E-06	0.0394	5	WDR70	1.0	0.6
cg26109981	8.9E-06	0.0404	5	-	1.6	-0.3
cg17223624	9.2E-06	0.0411	19	GPR108	1.6	1.7
cg22105215	9.3E-06	0.0415	5	DTWD2	2.5	-0.1
cg04407474	9.7E-06	0.0428	1	ORC1L;PRPF38A	1.7	0.7
cg14087061	1.0E-05	0.0441	9	HAUS6	3.4	1.4
cg05065583	1.0E-05	0.0443	2	CLK1	1.1	0.3
