# Supplementary material for: The effects of long-term daily folic acid and vitamin B12 supplementation on genome-wide DNA methylation in elderly subjects
Source: Clin Epigenetics. 2015 Nov 14;7:121. doi: 10.1186/s13148-015-0154-5 (PMC4644301; doi:10.1186/s13148-015-0154-5)
Supplement: Additional file 3: Table S2a and S2b. — Differentially methylated positions and regions related to serum levels of folate, vitamin B12 of plasma homocysteine. Overview of the top-10 of a) differentially methylated positions as well as b) regions related to serum levels of folate, vitamin B12 of plasma homocysteine. [file 13148_2015_154_MOESM3_ESM.pdf]

Supplementary Table S2: Differentially methylated positions and regions related to serum levels of folate, vitamin B12 or plasma homocysteine. Overview of the top-10 of **A)** differentially methylated positions as well as **B)** regions related to serum levels of folate, vitamin B12 or plasma homocysteine.

**A)** Differentially methylated positions						
		P-value	chr	CpG density	Gene(s)	Gene feature
**Serum folate**						
1	cg00063734	1.88E-06	15	S_Shore	-	IGR
2	cg17054900	3.49E-06	3	S_Shore	DHX36	TSS1500
3	cg04138591	4.35E-06	7	Island	-	IGR
4	cg18630811	7.05E-06	19	OpenSea	ILF3	Body
5	cg16489826	7.24E-06	6	Island	TCP1; MRPL18	TSS1500;TSS200
6	cg13089318	1.04E-05	1	OpenSea	-	IGR
7	cg16101101	1.11E-05	1	Island	ILF2	TSS200
8	cg20143955	1.23E-05	1	OpenSea	TARS2	TSS200
9	cg11588710	1.27E-05	3	OpenSea	-	IGR
10	cg06860864	1.42E-05	6	S_Shore	-	IGR
**Serum vitamin B12**						
1	cg05180471	2.34E-06	6	Island	BAT5	TSS200
2	cg01334229	3.90E-06	3	OpenSea	-	IGR
3	cg13801712	4.18E-06	1	Island	RRAGC	TSS1500
4	cg03120115	8.67E-06	19	N_Shelf	ZNF347	5'UTR
5	cg24792272	8.93E-06	8	Island	MCM4; PRKDC	TSS1500;TSS1500
6	cg06073402	1.02E-05	17	Island	DBF4B	5'UTR;1stExon
7	cg19869971	1.40E-05	1	OpenSea	-	IGR
8	cg05805987	1.47E-05	8	Island	EIF3E	TSS200
9	cg01459962	1.55E-05	6	Island	CCDC101	TSS200
10	cg11079354	1.64E-05	6	OpenSea	BAT5	TSS200
**Plasma homocysteine**						
1	cg16002929	6.61E-07	5	OpenSea	SEMA5A	Body
2	cg11692577	1.32E-06	11	OpenSea	-	IGR
3	cg23366972	1.56E-06	21	S_Shore	C21orf2	TSS1500
4	cg00601648	1.54E-05	8	OpenSea	ADCY8	Body
5	cg04247967	1.97E-05	12	N_Shelf	-	IGR
6	cg04905210	2.21E-05	8	N_Shore	POLR3D	TSS1500
7	cg21456083	2.23E-05	19	S_Shore	-	IGR
8	cg04964676	2.78E-05	6	OpenSea	-	IGR
9	cg18485955	3.42E-05	16	OpenSea	TNFRSF17	TSS1500
10	cg21876145	3.55E-05	2	OpenSea	LAPTM4A	Body

**B)** Differentially methylated regions (DMRs)							
		Gene feature(s)	Chr	hg19 coordinates	probes	minpval	meanpval
**Serum folate (10/173 DMRs)**							
1	PDPK1,LOC652276	3'UTR,TSS1500,TSS200,Body	16	2652696-2653987	12	8.1E-07	1.2E-05
2	HOXA4	1stExon,5'UTR,TSS200,TSS1500	7	27169957-27171401	22	5.9E-06	1.5E-03
3	VWA5B2,MIR1224	Body,TSS200,3'UTR	3	183959000-183959853	11	1.1E-05	1.3E-04
4	TMEM194A	Body,1stExon,5'UTR,TSS200,TSS1500	12	57472074-57472858	13	3.6E-05	3.4E-04
5	-	-	5	8457548-8458392	7	4.7E-05	1.2E-04
6	ALKBH6,CLIP3	Body,5'UTR,1stExon,TSS200,3'UTR,TSS1500	19	36504494-36505654	9	8.7E-05	1.4E-03
7	TOP2A	Body,5'UTR,1stExon,TSS200,TSS1500	17	38573776-38574599	16	9.5E-05	5.4E-03
8	GNB2L1,SNORD96A,SNORD95	Body,TSS1500,TSS200,1stExon,5'UTR	5	180670058-180671419	23	9.8E-05	1.2E-03
9	BRCA1,NBR2	5'UTR,Body,TSS1500,1stExon,TSS200	17	41276606-41277847	22	1.0E-04	2.3E-03
10	C2orf63,RPS27A	5'UTR,TSS1500,TSS200,1stExon,Body	2	55458694-55460405	21	1.2E-04	7.0E-03
**Serum vitamin B12 (10/425 DMRs)**							
1	RNF5,AGPAT1,RNF5P1	TSS1500,5'UTR,1stExon,TSS200,Body	6	32144667-32146779	37	6.5E-06	2.9E-03
2	TAGLN	TSS1500,TSS200,5'UTR,1stExon	11	117069479-117070485	7	6.5E-06	2.2E-05
3	PLD6	Body,1stExon,5'UTR,TSS200,TSS1500	17	17108846-17110353	16	2.6E-05	9.6E-04
4	C1orf109,CDCA8	1stExon,5'UTR,TSS200,TSS1500	1	38156178-38157567	15	4.4E-05	1.0E-02
5	PPP4C	TSS1500,TSS200,5'UTR,1stExon,Body	16	30086175-30087955	12	4.5E-05	6.9E-03
6	RRAGC	1stExon,TSS200,TSS1500	1	39325107-39325951	11	4.6E-05	3.9E-03
7	LOC149837	Body,TSS200,TSS1500	20	5485144-5485873	9	7.4E-05	1.9E-04
8	TRIM4	Body,1stExon,5'UTR,TSS200,TSS1500	7	99516603-99517509	14	7.5E-05	2.1E-04
9	-	-	1	113286397-113287238	6	8.0E-05	8.7E-04
10	SNORD84,BAT1	Body,5'UTR,TSS1500,1stExon,TSS200	6	31508916-31510491	34	8.2E-05	3.3E-03
**Plasma homocysteine (10/11 DMRs)**							
1	HM13,PSIMCT-1	Body,TSS1500,TSS200	20	30134188-30135362	11	6.3E-11	4.5E-07
2	HLA-DPB1	Body	6	33047944-33049360	21	4.0E-10	1.5E-04
3	-	-	8	1708438-1708526	3	3.4E-04	3.9E-04
4	-	-	20	62570072-62570696	3	4.0E-04	6.1E-04
5	TRIM6-TRIM34,TRIM6	TSS1500,TSS200,1stExon,5'UTR	11	5617273-5617926	7	9.3E-04	5.5E-03
6	-	-	6	29895016-29895260	9	1.6E-03	1.3E-02
7	LMF1	Body	16	979662-980146	5	9.7E-03	1.8E-02
8	IL17RE	1stExon,5'UTR	3	9944512-9944537	2	1.1E-02	1.2E-02
9	NOTCH4	Body	6	32191210-32191496	4	2.8E-02	3.0E-02
10	-	-	2	74347616-74347776	2	4.1E-02	4.2E-02

For these analyses all 174 samples (n=172 for homocysteine because of 2 missing values) samples were included (i.e. baseline and follow-up samples for all 87 participants). In case no gene(s) is presented the specific differentially methylated position or region referred to an intergenic region. DMRs in Table 2b were identified using the DMRcate package available through Bioconductor. DMRs were sorted and presented based on the minimal p-value for all of the probes within the specific DMR. Abbreviations: IGR: intergenic region, DMR: differentially methylated region, h19: coordinates for the human genome based on the Genome Reference Consortium Human Build 37 (hg19/GRCh37), Chr: chromosome, TSS200: 200 base pairs around the transcription start site, TSS1500: 1500 base pairs around the transcription start site, 3’UTR: 3’ untranslated region, 5’UTR: 5’untranslated region.
